# Supplementary figures and images for: Resistance to pentamidine is mediated by AdeAB, regulated by AdeRS, and influenced by growth conditions in Acinetobacter baumannii ATCC 17978
Source: PLoS One. 2018 May 11;13(5):e0197412. doi: 10.1371/journal.pone.0197412 (PMC5947904; doi:10.1371/journal.pone.0197412)

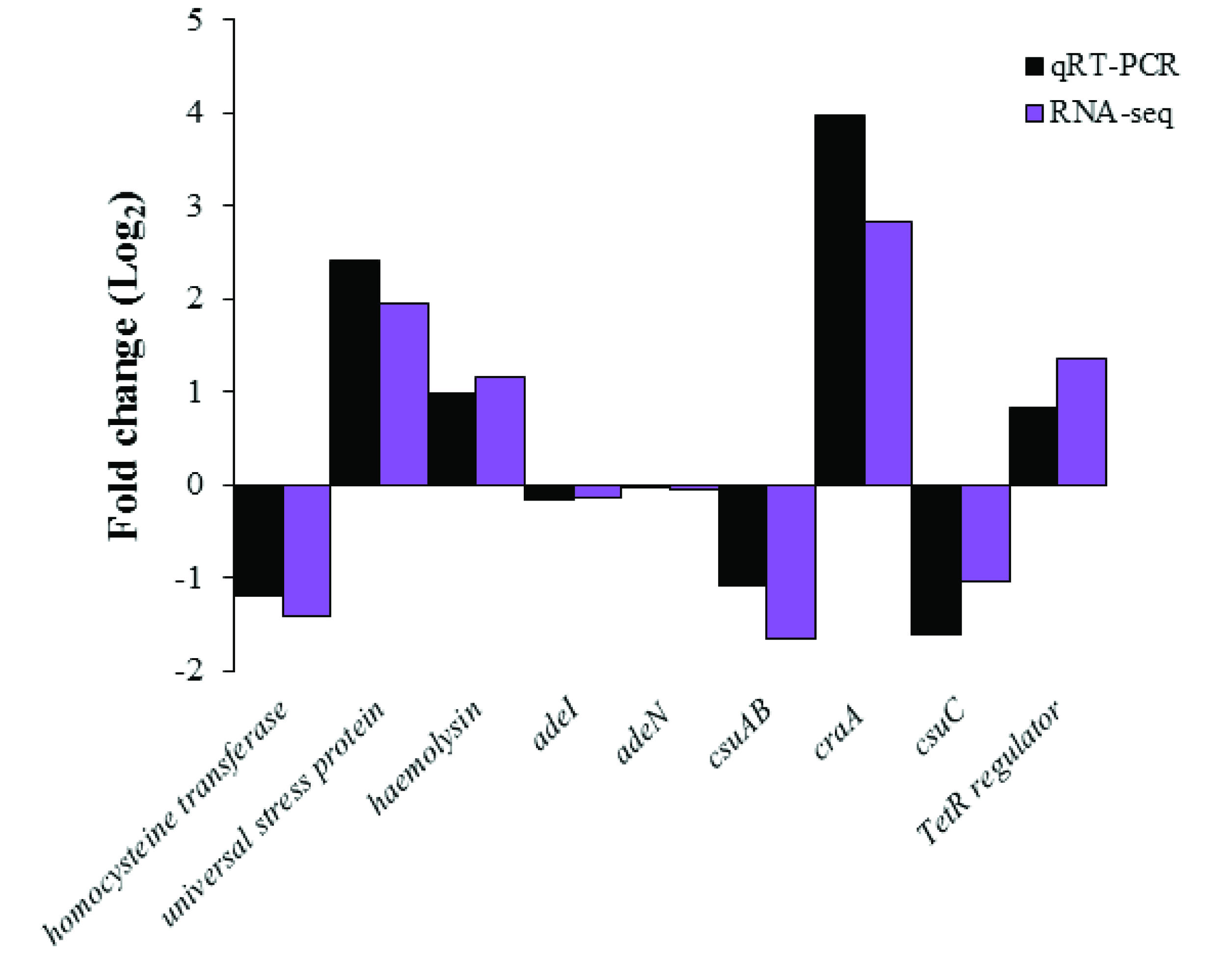

Supplement: S1 Fig — The transcriptomic results obtained by RNA-sequencing were validated by qRT-PCR analysis. The level of nine genes that displayed differential expression or remained essentially unchanged between ΔadeRS and WT ATCC 17978 were chosen for comparison. Expression levels for qRT-PCR experiments were corrected to those obtained for GAPDH (ACX60_05065) prior to normalisation against WT ATCC 17978 transcriptional levels. Grey and black bars represent values obtained from RNA-seq and qRT-PCR results, respectively. Differential expression between ΔadeRS and WT ATCC 17978 are given in Log2-values. (TIF) [file pone.0197412.s001.tif]

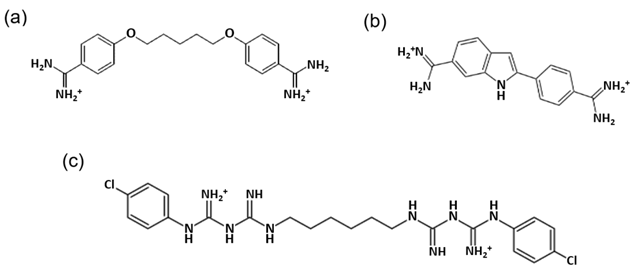

Supplement: S2 Fig — Compounds include (a) pentamidine, (b) DAPI and (c) chlorhexidine. For pentamidine and chlorhexidine, the cationic nitrogenous groups are separated by a long carbon chain, forming symmetrical compounds, whereas, DAPI lacks this long linker and is asymmetric. (TIF) [file pone.0197412.s002.tif]

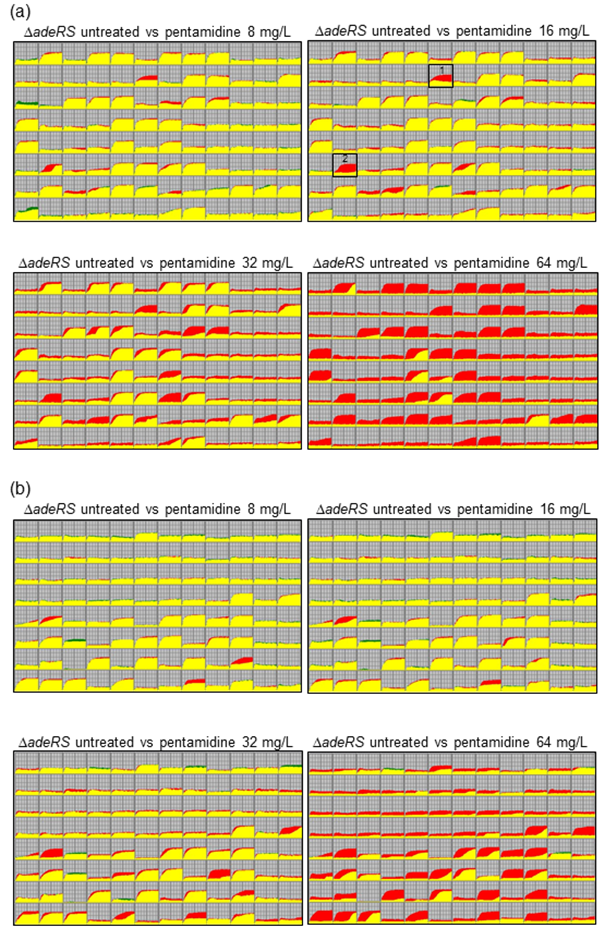

Supplement: S3 Fig — Respiration of ATCC 17978 ΔadeRS cells in the presence of pentamidine (8, 16, 32, or 64 mg/L) against untreated control cells are shown for (a) Biolog PM01 plates and (b) Biolog PM02A plates. Respiration activity for both plates were monitored in IF-0 (Biolog, Inc.) liquid medium for 72 h at 37°C. The curve in each well represents the colour intensity of a redox-active dye (y axis) over time (x axis: 72 h). Respiration of ΔadeRS cells are shown in red (control), green (under different concentrations of pentamidine), and yellow (depicts the regions of respiratory overlap). Black numbered squares represent carbon sources which decreased resistance to pentamidine (1, D-gluconic acid; 2, citric acid). (TIF) [file pone.0197412.s003.tif]

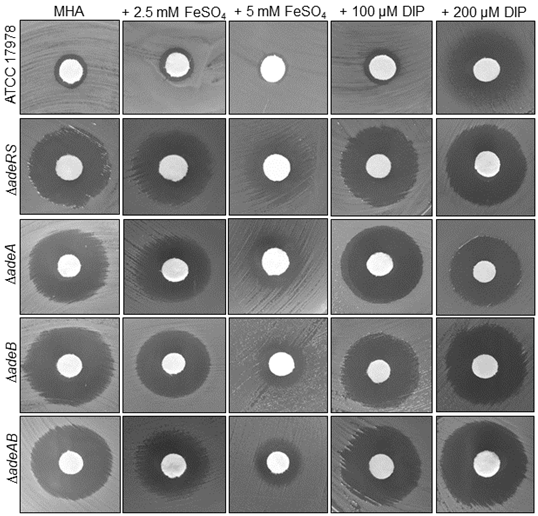

Supplement: S4 Fig — Resistance to pentamidine was assessed by disc diffusion assays in Mueller-Hinton agar (MHA) for ATCC 17978 and ΔadeRS, ΔadeA, ΔadeB and ΔadeAB deletion derivatives. Zones of clearing were compared to iron rich conditions from the addition of ferrous sulphate (FeSO4) at the final concentrations of 2.5 and 5 mM and iron-chelated conditions obtained by the addition of 2’,2’ dipyridyl (DIP) at the final concentrations of 100 and 200 μM in MHA. Images displayed are a representative of the typical results obtained. (TIF) [file pone.0197412.s004.tif]
