# Supplementary material for: Resistance to pentamidine is mediated by AdeAB, regulated by AdeRS, and influenced by growth conditions in Acinetobacter baumannii ATCC 17978
Source: PLoS One. 2018 May 11;13(5):e0197412. doi: 10.1371/journal.pone.0197412 (PMC5947904; doi:10.1371/journal.pone.0197412)
Supplement: S1 Table — (DOCX) [file pone.0197412.s005.docx]

**Table S1. Strains and plasmids used in the study**

| Strain or plasmid | Relevant characteristics^a^ | Reference/Source |
| --- | --- | --- |
| *A. baumannii* strains | | |
| ATCC 17978 | Non-international type clone; Meninges isolate | ATCC [[1](#_ENREF_1)] |
| ∆*adeRS* | ATCC 17978 with insertion disruption in *adeRS* | This study |
| ∆*adeAB* | ATCC 17978 with insertion disruption in *adeAB* | This study |
| ∆*adeA* | ATCC 17978 with insertion disruption in *adeA* | This study |
| ∆*adeB* | ATCC 17978 with insertion disruption in *adeB* | This study |
| ∆*adeRS* pWH::*adeRS* | ∆*adeRS* harboring pWH::*adeRS* | This study |
| ∆*adeRS* pWH1266 | ∆*adeRS* harboring pWH1266 | This study |
| ∆*adeRS* pWH::*adeAB* | ∆*adeRS* harboring pWH::*adeAB* | This study |
| ∆*adeRS* pWHgent::*adeAB* | ∆*adeRS* harboring pWHgent::*adeAB* | This study |
| ∆*adeRS* pWHgent | ∆*adeRS* harboring pWHgent | This study |
| ∆*adeA* pWHgent::*adeAB* | ∆*adeA* harboring pWHgent::*adeAB* | This study |
| ∆*adeA* pWHgent | ∆*adeA* harboring pWHgent | This study |
| ∆*adeB* pWHgent::*adeAB* | ∆*adeB* harboring pWHgent::*adeAB* | This study |
| ∆*adeB* pWHgent | ∆*adeB* harboring pWHgent | This study |
| ∆*adeAB* pWHgent::*adeAB* | ∆*adeAB* harboring pWHgent::*adeAB* | This study |
| ∆*adeAB* pWHgent | ∆*adeAB* harboring pWHgent | This study |
| *E. coli* strains | | |
| DH5α | F^–^ Φ80lacZΔM15 Δ(lacZYA-argF) U169 recA1 endA1 hsdR17 (rK^–^, mK^+^) phoA supE44 λ^–^ thi-1 gyrA96 relA1 | [[2](#_ENREF_2)] |
| Plasmids | | |
| pAT04 | TET^R^; pMMB67EH with Rec_Ab_ system | [[3](#_ENREF_3)] |
| pBluescript SK^+^ II | AMP^R^; Cloning vector | [[4](#_ENREF_4)] |
| pBl_-*Bam*HI | AMP^R^; pBluescript SK^+^ II with *Bam*HI restriction site removed via end-filling | This study |
| pBl_*adeRS* | AMP^R^; pBl_-*Bam*HI with *adeRS* flanking regions and ERY resistance cassette cloned via *Xba*I | This study |
| pEX18Tc | TET^R^; *sacB*-based suicide vector | [[5](#_ENREF_5)] |
| pEX_*adeRS* | TET^R^, Ery^R^; pEX18Tc with *adeRS* flanking regions and ERY resistance cassette cloned via *Xba*I | This study |
| pUCGM | GEN^R^; Source of GEN resistance cassette | [[6](#_ENREF_6)] |
| pVA891 | CHL^R^, ERY^R^; Source of ERY resistance cassette | [[7](#_ENREF_7)] |
| pWH1266 | AMP^R^, TET^R^; *Acinetobacter*/ *E. coli* shuttle vector | [[8](#_ENREF_8)] |
| pWHgent | AMP^R^, GEN^R^; pWH1266 with GEN resistance cassette cloned via *Bam*HI | This study |
| pWH::*adeRS* | AMP^R^; pWH1266 with *adeRS* cloned via *Bam*HI and *Sal*I | This study |
| pWH::*adeAB* | AMP^R^; pWH1266 with *adeAB* cloned via *Bam*HI and *Sph*I | This study |
| pWHgent::*adeAB* | AMP^R^, Gent^R^; pWHgent with *adeAB* cloned via *Bam*HI and *Sph*I | This study |

^a^AMP, ampicillin; CHL, chloramphenicol; ERY, erythromycin; GEN, gentamicin; ^R^, resistant; TET, tetracycline

**References for Table S1**

1. Smith MG, Gianoulis TA, Pukatzki S, Mekalanos JJ, Ornston LN, Gerstein M, et al. New insights into *Acinetobacter baumannii* pathogenesis revealed by high-density pyrosequencing and transposon mutagenesis. Genes Dev. 2007;21(5):601-14.

2. Hanahan D. Studies on transformation of *Escherichia coli* with plasmids. J Mol Biol. 1983;166(4):557-80.

3. Tucker AT, Nowicki EM, Boll JM, Knauf GA, Burdis NC, Trent MS, et al. Defining gene-phenotype relationships in *Acinetobacter baumannii* through one-step chromosomal gene inactivation. MBio. 2014;5(4):e01313-14.

4. Alting-Mees MA, Short JM. pBluescript II: gene mapping vectors. Nucleic Acids Res. 1989;17(22):9494.

5. Hoang TT, Karkhoff-Schweizer RR, Kutchma AJ, Schweizer HP. A broad-host-range Flp-FRT recombination system for site-specific excision of chromosomally-located DNA sequences: application for isolation of unmarked *Pseudomonas aeruginosa* mutants. Gene. 1998;212(1):77-86.

6. Schweizer HD. Small broad-host-range gentamycin resistance gene cassettes for site-specific insertion and deletion mutagenesis. Biotechniques. 1993;15(5):831-4.

7. Macrina FL, Evans RP, Tobian JA, Hartley DL, Clewell DB, Jones KR. Novel shuttle plasmid vehicles for *Escherichia*-*Streptococcus* transgeneric cloning. Gene. 1983;25(1):145-50.

8. Hunger M, Schmucker R, Kishan V, Hillen W. Analysis and nucleotide sequence of an origin of DNA replication in *Acinetobacter calcoaceticus* and its use for *Escherichia coli* shuttle plasmids. Gene. 1990;87(1):45-51.
