# Supplementary material for: Resistance to pentamidine is mediated by AdeAB, regulated by AdeRS, and influenced by growth conditions in Acinetobacter baumannii ATCC 17978
Source: PLoS One. 2018 May 11;13(5):e0197412. doi: 10.1371/journal.pone.0197412 (PMC5947904; doi:10.1371/journal.pone.0197412)
Supplement: S2 Table — (DOCX) [file pone.0197412.s006.docx]

**Table S2 Primers used in this study**

| **Primer name/ purpose** | **Sequence (5’–3’)^a^** |
| --- | --- |
| **Primers used for construction of ∆*adeA,* ∆*adeB*, ∆*adeAB* and ∆*adeRS*** | |
| ∆*adeRS* | |
| *adeRS*_UFR_F^b^ | GAGATCTAGATACGCATAGCTTTCTCGGCACC |
| *adeRS*_UFR_R | GAGAGGATCCATCGTAGTCATCTTCTACCAC |
| *adeRS*_DFR_F | GAGAGGATCCCGCTATTTTCTGTTAGTAGTGGG |
| *adeRS*_DFR_R | GAGATCTAGAGCTCTTAAAAACAGTTACTC |
| Ery_*Bam*HI_F | GAGAGGATCCCTTAAGAGTGTGTTGATAGTGC |
| Ery_*Bam*HI_R | GAGAGGATCCCTCATAGAATTATTTCCTCCG |
| *adeRS*_check_F | GCAGCCGCGGTAGCAGGC |
| *adeRS*_check_R | GGGGTCAAACACAGACGAC |
|  |  |
| ∆*adeB* | |
| *adeB*_UFR_F | TTACCAATAGCCACGGGC |
| *adeB*_UFR_R | CTATCAACACACTCTTAAGGGCAGTTTAGGAATAC |
| *adeB*_DFR_F^b^ | CGGGAGGAAATAATTCTATGTCAGCCATTTATAGTC |
| *adeB*_DFR_R^b^ | GGCCAACGCTTAAATACAT |
| Ery_F | CTTAAGAGTGTGTTGATAGTGC |
| Ery_R | CTCATAGAATTATTTCCTCCG |
| *adeB*_NOL_F | CCTTATAACGTCACAGCA |
| *adeB*_NOL_R^b^ | CGGGTGGTGAGCGTC |
|  |  |
| ∆*adeAB* | |
| *adeAB*_UFR_F | GAGAGTCGACTGAGCTTAAACTAATCCAGCC |
| *adeA*_UFR_R^b^ | CTATCAACACACTCTTAAGGTCCAAACCTAGTGAGTTTTTG |
| *adeB*_DFR_F^b^ | CGGGAGGAAATAATTCTATGTCAGCCATTTATAGTC |
| *adeB*_DFR_R^b^ | GGCCAACGCTTAAATACAT |
| Ery_F | CTTAAGAGTGTGTTGATAGTGC |
| Ery_R | CTCATAGAATTATTTCCTCCG |
| *adeAB*_NOL_F^b^ | GCTATGAGTGTCGGTATCAATTT |
| *adeB*_NOL_R^b^ | CGGGTGGTGAGCGTC |
|  |  |
| ∆*adeA* | |
| *adeAB*_UFR_F | GAGAGTCGACTGAGCTTAAACTAATCCAGCC |
| *adeA*_UFR_R^b^ | CTATCAACACACTCTTAAGGTCCAAACCTAGTGAGTTTTTG |
| Ery_F | CTTAAGAGTGTGTTGATAGTGC |
| Ery_rev_*adeA* | TCATTTCCTCCCGTTAAATAATAG |
| *adeA*_DFR_F | CTATTATTTAACGGGAGGAAATGATGTCACAATTTTTTATTCG |
| *adeA*_DFR_R | CTTTCAATTGCATACGTG |
| *adeA*_NOL_F^b^ | GCTATGAGTGTCGGTATCAATTT |
| *adeA_*NOL*_*R^b^ | GAGATCTAGATACGCATAGCTTTCTCGGCACC |
| **Primers used for complementation of ∆*adeA,* ∆*adeB*, ∆*adeAB* and ∆*adeRS*** | |
| *adeAB*_comp_F | GAGAGGATCCATCGTAGTCATCTTCTACCAC |
| *adeAB*_comp_R | GAGAGCATGCGACTATAAATGGCTGAC |
| Gent_comp_F | GAGAGGATCCCGAATTGACATAAGCC |
| Gent_comp_R | GAGAGGATCCGCTTGAACGAATTGTT |
| *adeRS*_comp_F | GAGAGGATCCGTGTGGAGTAAGTGTGGAGA |
| *adeRS*_comp_R | GAGAGTCGACGCGAGAAGAGATTCGTAGAAG |
| **Primers used for qRT-PCR** | |
| 16S_RT_F^c^ | CAGCTCGTGTCGTGAGATGT |
| 16S_RT_R^c^ | CGTAAGGGCCATGATGACTT |
| *GAPDH*_ RT_F^c^ | CAACACTGGTAAATGGCGTG |
| *GAPDH*_ RT_R^c^ | ACAACGTTTTTCATTTCGCC |
| ACX60_17010_RT_F | CAAAAGCAAAAGCACCACAA |
| ACX60_17010_RT_R | GAAGAAGAATCTGGCCATGC |
| ACX60_15380_ RT_F | CCGTGAATGGATTTACAGTTTAGT |
| ACX60_15380_ RT_R | GGTTTGTTAATTGTCCCGTCA |
| ACX60_14705_ RT_F | TTGCCAAAATCTTGAACCAA |
| ACX60_14705_ RT_R | AGTCGCAATACCCCAGTCAT |
| ACX60_11550_RT_F | CGTGATAATCAGGCGAACTG |
| ACX60_11550_RT _R | GGTTGACCTGGAGCAACTTT |
| ACX60_07895_RT_F | CATGCTGGTGGTTCAAAAAC |
| ACX60_07895_RT_R | GCTCTGGTTGAAATGCAATG |
| *csuC*_RT_F | GTGGATTAACCGAAGAAAGTCA |
| *csuC*_RT_R | GGCTGGCCTTGTTGATTG |
| *csuAB*_RT_F | GGTGAACGTACAGACCGCA |
| *csuAB*_RT_R | AGTAGCTTGGCCACTTACTGTAGT |
| *adeI*_RT_F | AATTGTTCAGGGCGTTGTTC |
| *adeI*_RT_R | GTTTCAACAGGACGGCTCTC |
| *craA*_RT_F | CGGCAGTTCCTTGGGTTA |
| *craA*_RT_R | AACCATATTGCACGCTCGT |
| *adeA*_RT_F | AAGCTGAGGTGGCAAGACTC |
| *adeA*_RT_R | TGCTTTCATTTGAGCGACAT |
| *adeS*_RT_F | CGGCGACCTCTCTGCTAG |
| *adeS*_RT_R | ATGGCTGCATTCCAAACC |

^a^Underlined nucleotide bases identify incorporated restriction sites: TCTAGA, *Xba*I; GGATTC, *Bam*HI; GTCGAC, *Sal*I; GCATGC, *Sph*I

^b^Primers used for multiple functions

^c^Primers used to amplify genes used as controls/references for qRT-PCR experiments
