# Supplementary material for: Resistance to pentamidine is mediated by AdeAB, regulated by AdeRS, and influenced by growth conditions in Acinetobacter baumannii ATCC 17978
Source: PLoS One. 2018 May 11;13(5):e0197412. doi: 10.1371/journal.pone.0197412 (PMC5947904; doi:10.1371/journal.pone.0197412)
Supplement: S3 Table — (DOCX) [file pone.0197412.s007.docx]

**Table S3.** **Genes significantly up- and down-regulated (≥ 2-fold) in ∆*adeRS* compared against the parent ATCC 17978 (CP012004) by RNA-seq methodologies.**

| **Gene ID** | **Gene Annotation** | **Log_2_ fold change** |
| --- | --- | --- |
| **Genes significantly down-regulated** | | |
| ACX60_05645 | isochorismatase | -3.86 |
| ACX60_05640 | enterobactin synthase subunit E | -3.77 |
| ACX60_05605 | iron ABC transporter permease | -3.46 |
| ACX60_09665 | siderophore 1 biosynthesis protein | -2.68 |
| ACX60_05630 | RhbE rhizobactin siderophore biosynthesis protein | -2.65 |
| ACX60_05600 | acinetobactin biosynthesis protein | -2.62 |
| ACX60_09120 | AdeR response regulator protein | -2.55 |
| ACX60_05655 | hypothetical protein | -2.51 |
| ACX60_09720 | siderophore 1 biosynthesis protein | -2.48 |
| ACX60_05680 | isochorismate synthase | -2.47 |
| ACX60_05615 | iron ABC transporter ATP-binding protein | -2.40 |
| ACX60_09710 | RND transporter | -2.33 |
| ACX60_05650 | histidine decarboxylase | -2.33 |
| ACX60_05710 | beta-lactamase | -2.17 |
| ACX60_05665 | ABC transporter | -2.06 |
| ACX60_09715 | ornithine monooxygenase | -1.97 |
| ACX60_05610 | iron ABC transporter permease | -1.96 |
| ACX60_05660 | ABC transporter | -1.95 |
| ACX60_00325 | 4-hydroxyphenylpyruvate dioxygenase | -1.94 |
| ACX60_17945 | alkanesulphonate monooxygenase | -1.91 |
| ACX60_06600 | C4-dicarboxylate transporter | -1.85 |
| ACX60_17950 | ABC transporter permease | -1.80 |
| ACX60_11680 | type VI secretion protein | -1.80 |
| ACX60_11955 | ATP-binding protein | -1.78 |
| ACX60_05620 | ferric anguibactin-binding protein | -1.75 |
| ACX60_06480 | CsuA/B protein | -1.66 |
| ACX60_16650 | DNA transfer protein p32 | -1.62 |
| ACX60_14710 | hypothetical protein | -1.57 |
| ACX60_09200 | transporter | -1.56 |
| ACX60_11455 | 2,2 C3-dehydroadipyl-CoA hydratase | -1.56 |
| ACX60_09250 | acetyl-CoA acetyltransferase | -1.53 |
| ACX60_11685 | hypothetical protein | -1.53 |
| ACX60_07755 | hypothetical protein | -1.51 |
| ACX60_05635 | peptide synthetase | -1.50 |
| ACX60_06500 | CsuD protein | -1.47 |
| ACX60_17735 | L-lactate permease | -1.47 |
| ACX60_17955 | aliphatic sulphonates transport ATP-binding subunit | -1.42 |
| ACX60_14705 | 5-methyltetrahydropteroyltriglutamate-- homocysteine methyltransferase | -1.42 |
| ACX60_05595 | peptide synthetase | -1.41 |
| ACX60_06705 | 30S ribosomal protein S18 | -1.38 |
| **Gene ID** | **Gene Annotation** | **Log_2_ fold change** |
| ACX60_09240 | succinyl-CoA:3-ketoacid-CoA transferase | -1.35 |
| ACX60_07070 | alcohol dehydrogenase | -1.34 |
| ACX60_11450 | enoyl-CoA hydratase | -1.33 |
| ACX60_06710 | 30S ribosomal protein S6 | -1.31 |
| ACX60_09115 | AdeS histidine kinase protein | -1.31 |
| ACX60_09315 | 4Fe-4S ferredoxin | -1.30 |
| ACX60_17240 | TonB-dependent receptor | -1.30 |
| ACX60_09685 | ligand-gated channel protein | -1.27 |
| ACX60_00345 | fumarylacetoacetase | -1.25 |
| ACX60_00340 | maleylacetoacetate isomerase | -1.23 |
| ACX60_09605 | hypothetical protein | -1.22 |
| ACX60_06505 | CsuE protein | -1.21 |
| ACX60_06700 | 50S ribosomal protein L9 | -1.21 |
| ACX60_00750 | ligand-gated channel protein | -1.20 |
| ACX60_10725 | ABC transporter permease | -1.20 |
| ACX60_11715 | ABC transporter | -1.17 |
| ACX60_07055 | aldehyde dehydrogenase | -1.16 |
| ACX60_12900 | arginine N-succinyltransferase | -1.16 |
| ACX60_10705 | monooxygenase | -1.16 |
| ACX60_09700 | siderophore 1 biosynthesis protein | -1.13 |
| ACX60_11475 | phenylacetate-CoA oxygenase | -1.13 |
| ACX60_09235 | succinyl-CoA:3-ketoacid-CoA transferase | -1.11 |
| ACX60_00335 | glyoxalase | -1.09 |
| ACX60_09690 | dimethylmenaquinone methyltransferase | -1.07 |
| ACX60_11675 | EvpB family type VI secretion protein | -1.07 |
| ACX60_09245 | short chain fatty acid transporter | -1.07 |
| ACX60_17290 | glutathione peroxidase | -1.06 |
| ACX60_00950 | acetate permease | -1.06 |
| ACX60_09705 | AcsC siderophore 1 achromobactin biosynthesis protein | -1.06 |
| ACX60_11950 | secretion protein HlyD | -1.05 |
| ACX60_02525 | 50S ribosomal protein L13 | -1.04 |
| ACX60_11665 | type VI secretion protein | -1.03 |
| ACX60_06495 | CsuC protein | -1.03 |
| ACX60_02110 | 50S ribosomal protein L2 | -1.03 |
| ACX60_15730 | ligand-gated channel protein | -1.02 |
| ACX60_07195 | porin | -1.02 |
| ACX60_00935 | hypothetical protein | -1.01 |
| ACX60_10810 | Na+:H+ dicarboxylate symporter | -1.01 |
| ACX60_02115 | 30S ribosomal protein S19 | -1.00 |
| ACX60_02105 | 50S ribosomal protein L23 | -1.00 |
| **Genes significantly up-regulated** | | |
| ACX60_01760 | CraA multidrug transporter protein | 2.83 |
| ACX60_03300 | hypothetical protein | 2.54 |
| ACX60_10085 | hypothetical protein | 2.28 |
| ACX60_08970 | hypothetical protein | 2.24 |
| ACX60_12490 | hypothetical protein | 2.08 |
| ACX60_11320 | threonine transporter RhtB | 2.03 |
| ACX60_05985 | tRNA-Arg | 1.95 |
| **Gene ID** | **Gene Annotation** | **Log_2_ fold change** |
| ACX60_17005 | sulphate transporter | 1.94 |
| ACX60_18810 | sulphate transporter | 1.94 |
| ACX60_17010 | universal stress protein | 1.94 |
| ACX60_18815 | universal stress protein | 1.94 |
| ACX60_10140 | hypothetical protein | 1.88 |
| ACX60_03305 | Fis family transcriptional regulator protein | 1.87 |
| ACX60_08745 | hypothetical protein | 1.83 |
| ACX60_09475 | hypothetical protein | 1.81 |
| ACX60_18655 | replication protein C | 1.74 |
| ACX60_08795 | hypothetical protein | 1.70 |
| ACX60_18230 | hypothetical protein | 1.68 |
| ACX60_07175 | hypothetical protein | 1.66 |
| ACX60_07230 | hypothetical protein | 1.66 |
| ACX60_10065 | hypothetical protein | 1.66 |
| ACX60_12555 | hypothetical protein | 1.66 |
| ACX60_18670 | hypothetical protein | 1.66 |
| ACX60_18515 | hypothetical protein | 1.63 |
| ACX60_17015 | hypothetical protein | 1.63 |
| ACX60_18820 | hypothetical protein | 1.63 |
| ACX60_00635 | hypothetical protein | 1.62 |
| ACX60_18075 | dual-action HEIGH metallo-peptidase | 1.60 |
| ACX60_08515 | polyketide cyclase | 1.59 |
| ACX60_09520 | energy transducer TonB | 1.53 |
| ACX60_11880 | hypothetical protein | 1.52 |
| ACX60_08875 | fumarylacetoacetate hydrolase | 1.46 |
| ACX60_17020 | hypothetical protein | 1.46 |
| ACX60_18825 | hypothetical protein | 1.46 |
| ACX60_18585 | hypothetical protein | 1.44 |
| ACX60_01135 | TetR family transcriptional regulator | 1.40 |
| ACX60_08665 | ThiJ thiamine biosynthesis protein | 1.40 |
| ACX60_12390 | hypothetical protein | 1.40 |
| ACX60_12550 | hypothetical protein | 1.40 |
| ACX60_06960 | hypothetical protein | 1.37 |
| ACX60_08965 | hypothetical protein | 1.37 |
| ACX60_10870 | ArsR family transcriptional regulator | 1.36 |
| ACX60_07215 | ABC transporter ATPase | 1.35 |
| ACX60_07685 | lysozyme | 1.35 |
| ACX60_15380 | TetR family transcriptional regulator | 1.35 |
| ACX60_06435 | membrane protein | 1.34 |
| ACX60_00085 | tRNA-Ala | 1.34 |
| ACX60_02440 | tRNA-Ala | 1.34 |
| ACX60_02605 | tRNA-Ala | 1.34 |
| ACX60_14995 | tRNA-Ala | 1.34 |
| ACX60_17210 | tRNA-Ala | 1.34 |
| ACX60_18015 | tRNA-Ala | 1.34 |
| ACX60_12795 | p-hydroxycinnamoyl CoA hydratase/lyase | 1.33 |
| ACX60_09040 | TetR family transcriptional regulator | 1.33 |
| ACX60_09125 | AdeA membrane protein | 1.33 |
| ACX60_12765 | MFS transporter | 1.32 |
| ACX60_08740 | bile acid:sodium symporter | 1.32 |
| ACX60_10155 | hypothetical protein | 1.32 |
| **Gene ID** | **Gene Annotation** | **Log_2_ fold change** |
| ACX60_00080 | tRNA-Ile | 1.31 |
| ACX60_02435 | tRNA-Ile | 1.31 |
| ACX60_02600 | tRNA-Ile | 1.31 |
| ACX60_15000 | tRNA-Ile | 1.31 |
| ACX60_17215 | tRNA-Ile | 1.31 |
| ACX60_18020 | tRNA-Ile | 1.31 |
| ACX60_02950 | hypothetical protein | 1.30 |
| ACX60_07185 | hypothetical protein | 1.30 |
| ACX60_07695 | hypothetical protein | 1.30 |
| ACX60_06535 | TetR family transcriptional regulator | 1.30 |
| ACX60_12085 | transposase | 1.30 |
| ACX60_07865 | hypothetical protein | 1.29 |
| ACX60_05560 | hypothetical protein | 1.28 |
| ACX60_04305 | molecular chaperone Tir | 1.28 |
| ACX60_08180 | acetyltransferase | 1.28 |
| ACX60_10175 | hypothetical protein | 1.27 |
| ACX60_04275 | GCN5 family acetyltransferase | 1.27 |
| ACX60_11730 | transcriptional regulator | 1.27 |
| ACX60_17650 | hypothetical protein | 1.26 |
| ACX60_07530 | hypothetical protein | 1.26 |
| ACX60_09725 | hypothetical protein | 1.25 |
| ACX60_12055 | cold-shock protein | 1.25 |
| ACX60_07860 | hypothetical protein | 1.25 |
| ACX60_00615 | hypothetical protein | 1.25 |
| ACX60_08150 | hypothetical protein | 1.25 |
| ACX60_09980 | hypothetical protein | 1.25 |
| ACX60_10070 | DNase | 1.25 |
| ACX60_10170 | hypothetical protein | 1.25 |
| ACX60_16000 | translation initiation factor IF-1 | 1.24 |
| ACX60_13910 | Ferric uptake regulator (Fur) protein | 1.24 |
| ACX60_16755 | DNA-binding protein | 1.24 |
| ACX60_02290 | tRNA-Arg | 1.23 |
| ACX60_02295 | tRNA-Arg | 1.23 |
| ACX60_02310 | tRNA-Arg | 1.23 |
| ACX60_07750 | histidine kinase | 1.23 |
| ACX60_18150 | hypoxanthine phosphoribosyltransferase | 1.22 |
| ACX60_12430 | hypothetical protein | 1.22 |
| ACX60_09525 | hypothetical protein | 1.21 |
| ACX60_10225 | orotidine 5'-phosphate decarboxylase | 1.20 |
| ACX60_08710 | 3-oxoacyl-ACP reductase | 1.20 |
| ACX60_09945 | hypothetical protein | 1.20 |
| ACX60_06940 | membrane protein | 1.20 |
| ACX60_18235 | hypothetical protein | 1.20 |
| ACX60_08510 | IacB protein | 1.19 |
| ACX60_11825 | hypothetical protein | 1.19 |
| ACX60_13405 | rubredoxin | 1.19 |
| ACX60_08040 | sulphate transporter | 1.19 |
| ACX60_08755 | hypothetical protein | 1.18 |
| ACX60_12955 | hypothetical protein | 1.18 |
| ACX60_03395 | alkylphosphonate utilisation protein | 1.18 |
| ACX60_16225 | hypothetical protein | 1.17 |
| **Gene ID** | **Gene Annotation** | **Log_2_ fold change** |
| ACX60_07150 | hypothetical protein | 1.17 |
| ACX60_11405 | reverse transcriptase | 1.17 |
| ACX60_10160 | hypothetical protein | 1.17 |
| ACX60_08760 | membrane protein | 1.16 |
| ACX60_08630 | FAH family protein | 1.16 |
| ACX60_11230 | hypothetical protein | 1.16 |
| ACX60_11550 | hemolysin | 1.16 |
| ACX60_03630 | hypothetical protein | 1.16 |
| ACX60_09055 | hypothetical protein | 1.15 |
| ACX60_07690 | hypothetical protein | 1.15 |
| ACX60_02580 | membrane protein | 1.15 |
| ACX60_01640 | fimbrial protein | 1.15 |
| ACX60_18225 | hypothetical protein | 1.15 |
| ACX60_10005 | hypothetical protein | 1.15 |
| ACX60_03480 | tRNA-Val | 1.14 |
| ACX60_03495 | tRNA-Val | 1.14 |
| ACX60_03505 | tRNA-Val | 1.14 |
| ACX60_11170 | AsnC family transcriptional regulator | 1.14 |
| ACX60_06565 | acetyltransferase | 1.14 |
| ACX60_14685 | Darcynin 1 | 1.13 |
| ACX60_02700 | hypothetical protein | 1.13 |
| ACX60_01625 | DNA-directed RNA polymerase subunit | 1.13 |
| ACX60_09855 | 30S ribosomal protein S20 | 1.12 |
| ACX60_08125 | hypothetical protein | 1.12 |
| ACX60_09990 | hypothetical protein | 1.12 |
| ACX60_14670 | Darcynin 1 | 1.11 |
| ACX60_10130 | DNA helicase | 1.11 |
| ACX60_06925 | hypothetical protein | 1.11 |
| ACX60_07515 | hypothetical protein | 1.11 |
| ACX60_07210 | DNA breaking-rejoining protein | 1.11 |
| ACX60_09765 | HU transcriptional regulator α subunit | 1.10 |
| ACX60_04280 | hypothetical protein | 1.10 |
| ACX60_02940 | hypothetical protein | 1.09 |
| ACX60_06915 | hypothetical protein | 1.09 |
| ACX60_06360 | tRNA-Leu | 1.09 |
| ACX60_06370 | tRNA-Leu | 1.09 |
| ACX60_08065 | hypothetical protein | 1.09 |
| ACX60_12360 | hypothetical protein | 1.08 |
| ACX60_02945 | hypothetical protein | 1.08 |
| ACX60_10525 | FxsA cytoplasmic membrane protein | 1.08 |
| ACX60_18560 | hypothetical protein | 1.08 |
| ACX60_01150 | hypothetical protein | 1.08 |
| ACX60_06250 | cold-shock protein | 1.08 |
| ACX60_05480 | tRNA-Val | 1.08 |
| ACX60_10025 | HK97 gp10 family phage protein | 1.08 |
| ACX60_10090 | hypothetical protein | 1.08 |
| ACX60_10970 | hypothetical protein | 1.08 |
| ACX60_11185 | hypothetical protein | 1.08 |
| ACX60_11765 | urea carboxylase | 1.08 |
| ACX60_12535 | hypothetical protein | 1.08 |
| ACX60_12960 | TetR family transcriptional regulator | 1.08 |
| ACX60_01975 | hypothetical protein | 1.07 |
| **Gene ID** | **Gene Annotation** | **Log_2_ fold change** |
| ACX60_06430 | blue light sensor protein | 1.07 |
| ACX60_16110 | tRNA-Lys | 1.06 |
| ACX60_16115 | tRNA-Lys | 1.06 |
| ACX60_18780 | recombinase | 1.06 |
| ACX60_12835 | GntR family transcriptional regulator | 1.06 |
| ACX60_15560 | acyl carrier protein | 1.06 |
| ACX60_07380 | hypothetical protein | 1.06 |
| ACX60_07670 | hypothetical protein | 1.05 |
| ACX60_10405 | TetR family transcriptional regulator | 1.05 |
| ACX60_12450 | hypothetical protein | 1.04 |
| ACX60_18730 | phosphoglucosamine mutase | 1.04 |
| ACX60_12485 | phage-like protein | 1.04 |
| ACX60_10750 | hypothetical protein | 1.04 |
| ACX60_03935 | hypothetical protein | 1.04 |
| ACX60_04120 | hypothetical protein | 1.04 |
| ACX60_12685 | hypothetical protein | 1.04 |
| ACX60_12090 | tRNA-Met | 1.04 |
| ACX60_10570 | hypothetical protein | 1.03 |
| ACX60_12965 | hypothetical protein | 1.03 |
| ACX60_00730 | arsenate reductase | 1.03 |
| ACX60_10660 | transcriptional regulator | 1.03 |
| ACX60_18775 | hypothetical protein | 1.03 |
| ACX60_14690 | hypothetical protein | 1.03 |
| ACX60_18725 | transposase | 1.02 |
| ACX60_18580 | hypothetical protein | 1.02 |
| ACX60_11010 | malonate decarboxylase subunit beta | 1.02 |
| ACX60_04295 | HIT family hydrolase | 1.02 |
| ACX60_07275 | AceI efflux protein | 1.02 |
| ACX60_08785 | hypothetical protein | 1.02 |
| ACX60_03485 | tRNA-Asp | 1.02 |
| ACX60_03490 | tRNA-Asp | 1.02 |
| ACX60_03500 | tRNA-Asp | 1.02 |
| ACX60_16820 | transcriptional regulator | 1.02 |
| ACX60_06470 | hypothetical protein | 1.01 |
| ACX60_07650 | lipoprotein | 1.01 |
| ACX60_18565 | hypothetical protein | 1.01 |
| ACX60_18250 | hypothetical protein | 1.01 |
| ACX60_18220 | hypothetical protein | 1.01 |
| ACX60_12655 | carbon storage regulator | 1.01 |
| ACX60_13365 | hypothetical protein | 1.01 |
| ACX60_06365 | tRNA-Trp | 1.01 |
| ACX60_06375 | tRNA-Trp | 1.01 |
| ACX60_08800 | hypothetical protein | 1.01 |
| ACX60_15375 | hypothetical protein | 1.00 |
| ACX60_07575 | stress-responsive nuclear envelope protein | 1.00 |
| ACX60_02830 | hypothetical protein | 1.00 |
| ACX60_12355 | hypothetical protein | 1.00 |
| ACX60_10595 | hypothetical protein | 1.00 |
| ACX60_07630 | hypothetical protein | 1.00 |
