# Supplementary material for: Resistance to pentamidine is mediated by AdeAB, regulated by AdeRS, and influenced by growth conditions in Acinetobacter baumannii ATCC 17978
Source: PLoS One. 2018 May 11;13(5):e0197412. doi: 10.1371/journal.pone.0197412 (PMC5947904; doi:10.1371/journal.pone.0197412)
Supplement: S4 Table — (DOCX) [file pone.0197412.s008.docx]

**Table S4. Zones of clearing obtained from growth on M9 minimal medium with the addition of different carbon sources after exposure to pentamidine**

| **Zone of clearing (mm)^ab^** | | | | | |
| --- | --- | --- | --- | --- | --- |
| **Strain** | **WT** | **∆*adeRS*** | **∆*adeAB*** | **∆*adeA*** | **∆*adeB*** |
| **M9 Minimal Medium** |  |  |  |  |  |
| + glucose | 1.8 ± 0.2 | 5.2 ± 0.1 | 5.8 ± 0.4 | 6.2 ± 0.6 | 6.9 ± 0.3 |
| + citric acid | 2.7 ± 0.8 | 5.4 ± 0.8 | 5.1 ± 0.4 | 5.1 ± 0.5 | 6.0 ± 0.2 |
| + isocitric acid | 1.2 ± 0.3 | 5.7 ± 0.1 | ND^c^ | ND | ND |
| + α-ketoglutaric acid | 0 ± 0 | 0 ± 0 | 0 ± 0 | 0 ± 0 | 0 ± 0 |
| + succinic acid | 0 ± 0 | 0 ± 0 | 0 ± 0 | 0 ± 0 | 0 ± 0 |
| + fumaric acid | 0 ± 0 | 0 ± 0 | 0 ± 0 | 0 ± 0 | 0 ± 0 |
| + malic acid | 0.9 ± 0.4 | 5.4 ± 0.5 | ND | ND | ND |
| + oxaloacetic acid | 1.2 ± 0.3 | 1.6 ± 0.5 | 1.7 ± 0.5 | 1.6 ± 0.6 | 1.7 ± 0.5 |
| + pyruvic acid | 1.3 ± 0.1 | 4.4 ± 1.3 | ND | ND | ND |
| + glutamic acid | 1.1 ± 0.2 | 5.4 ± 0.2 | ND | ND | ND |

^a^Zones of clearing determined on M9 minimal medium with each carbon source used at a final concentration of 0.4 %. Pentamidine (125 µg) was deposited onto a paper disc (5 mm diameter) and zones of growth inhibition determined after overnight incubation at 37 °C

^b^Average dimension of inhibition zones was determined by obtaining the diameter from the edge of bacterial growth to the disc (mm), ± represents standard deviation values determined from averages obtained from at least three independent experiments undertaken in duplicate

**^c^**ND, not done
